# Performance comparison of three commercial multiplex molecular panels for respiratory viruses at a South African academic hospital

**DOI:** 10.4102/ajlm.v13i1.2415

**Published:** 2024-08-20

**Authors:** Clinton van der Westhuizen, Mae Newton-Foot, Pieter Nel

**Affiliations:** 1 Division of Medical Microbiology, Department of Pathology, Faculty of Medicine and Health Sciences, Stellenbosch University, Cape Town, South Africa; 2 Department of Medical Microbiology, Tygerberg Hospital, National Health Laboratory Service, Cape Town, South Africa

**Keywords:** composite reference standard, multiplex reverse transcription polymerase chain reaction, molecular panels, respiratory viruses, Anyplex™ II RV16, FilmArray^®^ Respiratory 2.1 *plus* Panel, QIAstat-Dx^®^ Respiratory SARS-CoV-2 Panel

## Abstract

**Background:**

Respiratory infections are a major contributor to hospital admissions. Identification of respiratory pathogens by means of conventional culture and serology methods remains challenging. Multiplex molecular assays are an appealing alternative that endeavours to be rapid, more accurate and less arduous.

**Objective:**

The study aimed to compare the clinical performance of three commercial multiplex molecular assays for respiratory viruses.

**Methods:**

Forty-eight respiratory specimens obtained from patients at Tygerberg Hospital in the Western Cape province of South Africa were studied. These specimens were collected between May 2020 and August 2020. The results of the Seegene Anyplex™ II RV16, FilmArray^®^ Respiratory 2.1 *plus* Panel (FARP), and QIAstat-Dx^®^ Respiratory SARS-CoV-2 Panel (QRP) were analysed based on the overlapping targets. A composite reference standard was applied to provide a standard reference for comparison.

**Results:**

The overall sensitivity of the Seegene Anyplex™ II RV16 was 96.6% (57/59), the FARP 98.2% (56/57) and the QRP 80.7% (46/57). The overall specificities were 99.8% (660/661), 99.0% (704/711) and 99.7% (709/711), respectively. The QRP failed to detect coronaviruses and parainfluenza viruses in 41.7% (5/12) and 28.6% (4/14) of positive specimens, respectively, while the FARP produced the lowest target specificity of 88.4% (38/43) for rhinovirus/enterovirus.

**Conclusion:**

The overall specificity of all three platforms was comparable; however, the sensitivity of the QRP was inferior to that of the ARV and FARP.

**What this study adds:**

This study adds to the body of performance characteristics described for respiratory multiplex panels, especially in the African context where molecular diagnostics for infectious diseases are gaining momentum.

## Introduction

Respiratory infections are a major contributor to hospital admissions and result in considerable morbidity and mortality.^1,2^ Published literature denotes viruses and bacteria as the main aetiologies of such disease, and also emphasises the value of laboratory confirmation of specific causative pathogens.^3,4^ However, isolating and identifying the vast spectrum of infective microbes by means of conventional testing methods can be arduous.^5,6^ Adopting an approach of syndromic testing has thus garnered momentum since the first such test was approved by the Food and Drug Administration of the United States in 2009.^7^ Multiplex molecular assays offer this attractive diagnostic alternative, proving to be quicker and less labour-intensive, while being able to detect multiple targets simultaneously.^8^ The ability to identify respiratory pathogens both rapidly and accurately relates to multiple potential benefits, including decreased length of hospital stay, improved antimicrobial stewardship and seasonal outbreak surveillance.^4,7,9,10,11^ However, an important limitation to nucleic acid assay tests is the clinical relevance of the results – a positive result reflects the presence of an infective virus or its antigens, but it does not distinguish between present or past infection.^10^ Other potential drawbacks include false positive (FP) results due to cross-reactivity or nonspecific amplification, false negative (FN) results caused by preferential amplification of one target over another, and the high cost of commercial products.^10,11^ The routine use of multiplex polymerase chain reaction (PCR) platforms for the detection of respiratory pathogens in South Africa, and similarly in Africa, is uncertain, and there is a paucity of published research with little local data that contribute to diagnostic and treatment guidelines.^12^ The performance of a nucleic acid test must be evaluated before it is implemented for clinical use. The advent of coronavirus disease 2019, caused by the novel severe acute respiratory syndrome coronavirus 2 (SARS-CoV-2), has placed reference standards that are used to validate multiplex molecular assays under scrutiny.^13,14^ The composite reference standard (CRS) has been proposed as a reasonable method for this purpose, especially when no gold standard is available, and several imperfect tests are accessible to serve as references.^15^ A CRS combines multiple independent testing methods or approaches to establish the most accurate and reliable result for a particular measurement or diagnosis. It minimises the limitations and biases that may be present in any single individual method, and ensures reproducibility of results.^16,17^ However, as the sensitivity of the CRS increases with more component tests, the specificity may reciprocally decrease, which can lead to accuracy estimates for the index test that are biased.^17^

Tygerberg Hospital, an academic hospital in South Africa, employed the Anyplex™ II RV16 (ARV) at the time of this study to detect respiratory viruses. Patients that presented with upper respiratory symptomatology were investigated by means of this commercial assay to diagnose viral pathogens. However, both the BioFire^®^ FilmArray^®^ Respiratory Panel 2.1 *plus* (FARP) and the QIAstat-Dx^®^ Respiratory SARS-CoV-2 Panel (QRP) were already in use in the private health sector. These commercially available kits promised quicker turn-around times, with the addition of detecting bacterial pathogens. Other multiplex assays, for example xTAG^®^ Respiratory Pathogen Panel, eSensor^®^ Respiratory Viral Panel and Verigene^®^ Respiratory Virus Plus Nucleic Acid Test, are not commonly used in the South African laboratory landscape.

Implementation of molecular systems for clinical diagnosis has expanded considerably and, although the local epidemiology of respiratory pathogens has been described, published data regarding the evaluation of multiplex molecular assays for respiratory specimens in sub-Saharan Africa remain limited.^18,19,20^ This study aimed to compare the clinical performance of the ARV, FARP and QRP on respiratory specimens in a tertiary hospital setting in South Africa by applying a CRS.

## Methods

### Ethical considerations

Ethical approval for this study was obtained from the Health Research Ethics Committee of Stellenbosch University with study approval number, S22/03/040_Sub-study N20/04/047. A waiver of informed consent was obtained as routine patient results were anonymised and no further clinical procedures involving patients were performed for this study. Depersonalisation of data was done for the evaluation by allocating unique identifiers to the specimens. These identifiers were used to generate an anonymised entry into an electronic database. No personal details or laboratory information system indicators were captured on the database.

### Study design

This study performed the FARP and QRP on stored residual clinical respiratory specimens from an academic hospital. The ARV results were obtained from medical records containing previous routine testing of the same specimens at the hospital. To allow for the interpretation of sensitivity and specificity of each platform, a CRS was utilised to construct a reference standard for comparison between the three assays.

### Study assays

The ARV is developed by Seegene^®^ (Seoul, South Korea) and utilises tagging oligonucleotide cleavage and extension technology for simultaneous detection of melt curves from 16 viral targets.^21^ Pre-extraction and pre-reverse transcription are required, but it makes use of common real-time PCR platforms. A range of respiratory samples including sputum, bronchoalveolar lavage and tracheal aspirates, can be processed, and a final result is available within 2 h.

The FARP by bioMérieux^®^ BioFire^®^ (Marcy-l’Étoile, France) can detect 23 targets (19 viral and four bacterial) and makes use of melt curve-based reverse transcription PCR (RT-PCR) within a closed automated system.^22^ Respiratory specimens are inoculated in a single-use pouch which integrates nucleic acid extraction and a two-step multiplex RT-PCR to produce a result in 45 min.

The QRP from Qiagen^®^ (Hilden, Germany) not only detects 19 viruses and 3 bacteria, but also provides semi-quantitative cycle threshold values.^23^ The DiagCORE^®^ technology includes silica membrane-based nucleic acid extraction and highly sensitive RT-PCR. It has been designed to process dry nasopharyngeal swab specimens as well as transport liquid specimens by means of a single-use cartridge. This automated platform has a turn-around time of 69 min.

A few differences regarding the ARV, FARP and QRP targets must be noted (Online Supplementary Table 1). Firstly, the ARV only detects viral targets, it does not subtype influenza A (H1/H1-2009/H3) and does not test for coronavirus HKU1, Middle East respiratory syndrome coronavirus or SARS-CoV-2. Comparison of these viral targets in the other two assays, as well as the bacterial targets (*Bordetella pertussis, Bordetella parapertussis, Chlamydophila pneumoniae, Legionella pneumophila* and *Mycoplasma pneumoniae*), is thus not possible. Only the ARV can subtype respiratory syncytial virus into A or B and distinguish rhinovirus and enterovirus. The FARP does not detect bocavirus or *L. pneumophila*. Lastly, the QRP does not test for Middle East respiratory syndrome coronavirus, *B. parapertussis* or *C. pneumoniae*.

### Study specimens and processing

Forty-eight respiratory specimens (32 nasopharyngeal aspirates and 16 nasopharyngeal swabs in universal transport media) were included in this study based on results obtained through testing on the ARV. A maximum of approximately 50 tests could be considered for the study as resources for the FARP and QRP assays were limited. This routine testing occurred during 01 May 2020 and 31 August 2020 on patients that presented with upper respiratory illness to Tygerberg Hospital which is situated in the Western Cape province of South Africa. These clinical specimens were selected to only include targets that were represented across all three assays. Residual specimens were stored at −80 °C in microcentrifuge tubes after ARV testing. These specimens were thawed within a period of 12 months between 01 April 2021 and 30 May 2021 to be concurrently run on the FARP and QRP.

Nucleic acid extraction for the ARV was done via the bioMérieux^®^ NucliSENS^®^ easyMAG^®^ system (Marcy-l’Étoile, France). The ARV RT-PCR was performed on the Bio-Rad^®^ CFX96™ thermocycler (Redmond, Washington, United States). A test was considered valid if the amplicon was interpretable and the controls passed for that run. FARP and QRP processing were performed according to the manufacturers’ guidelines. A test was considered valid if it completed without error and the internal control passed. It remained valid in the instance where the internal control failed but a target was detected.

### Results interpretation

Where assays were unable to report subtype, identification to an appropriate group was considered acceptable – for example, influenza A H1 (FARP or QRP) was comparable to influenza A with no subtype (ARV). Where assays were able to distinguish between viruses, identification to an appropriate group was considered acceptable – for example, human rhinovirus (ARV) was comparable to rhinovirus/enterovirus (FARP and QRP).

Thawed specimens that failed to demonstrate targets on the FARP and QRP that were detected by the ARV were immediately re-run on the ARV, provided that these targets were used for comparative analysis. This allowed for parallel testing to account for nucleic acid degradation, specimen contamination, and errors in specimen labelling and/or storage. The repeat ARV results superseded the initial ARV results for analysis.

### Data analysis

A CRS was applied to the targets that were comparable across the assays. Therefore, each target that overlapped across all three assays was included in the CRS for that particular specimen. A true positive constituted agreement of two or more of the three assays. A true negative described no detection of targets by two or more of the three assays. An FP reflected a target detected by one assay, but a negative composite reference result. An FN indicated a target not detected by one assay, in contrast to a positive composite reference result.

The overall sensitivity and specificity of each platform were calculated based on the comparison of only overlapping targets according to the composite reference established. According to the acceptance criteria of the South African National Accreditation System, both sensitivity and specificity must exceed 90% to meet their standard for molecular testing;^24^ thus, ≥ 90% was used to indicate acceptable performance.

If comparison of a specific target was not possible, it was excluded from analysis. This included coronavirus HKU1, Middle East respiratory syndrome coronavirus, SARS-CoV-2, bocavirus, and all bacterial pathogens. As such, these targets could not form part of the CRS. McNemar’s chi-squared test was used to determine significant difference between the performance of an assay for a specific target and the CRS; a *p*-value of ≤ 0.05 was used to indicate significance.

### Results

Valid runs were obtained for all 48 specimens on both the FARP and QRP. One specimen failed initial testing on the QRP but was successful with an immediate repeat run, and another specimen had a QRP internal control failure but was deemed valid, as parainfluenza virus 3 was detected.

Thirteen specimens (27.1%) were positive for targets on the ARV that were not detected by either the FARP or QRP. These underwent repeat ARV testing and only two demonstrated loss of detectable targets (adenovirus and enterovirus, respectively) in comparison to the initial ARV result. As all three platforms failed to detect these targets, it was concluded that nucleic acid degradation was the most likely reason.

A composite reference was generated for all 48 specimens (Table 1). The overall sensitivity of the ARV (96.6%) and FARP (98.2%) was comparable. However, the QRP had a lower sensitivity of 80.7%. The three platforms had similar specificities ranging from 99.0% to 99.8% (Table 2).

**TABLE 1:** Seegene Anyplex™ II RV16, BioFire^®^ FilmArray^®^ Respiratory 2.1 *plus* Panel, and Qiagen^®^ QIAstat-Dx^®^ Respiratory SARS-CoV-2 Panel targets detected, and composite reference generated for 48 respiratory samples collected in South Africa during 2021 and 2022.

Specimen number	ARV	FARP	QRP	Repeat ARV	Composite reference
S1	ADV	RV/EV	None	None	None
S2	NL63	NL63	NL63	-	NL63
S3	MPV	MPV	MPV	-	MPV
S4	HRV	RV/EV	RV/EV	-	RV/EV
S5	FLUA	FLUA/H1-2009	FLUA/H1-2009	-	FLUA
S6	FLUA	ADV, FLUA/H1-2009	FLUA	-	FLUA
S7	FLUB	FLUB	FLUB	-	FLUB
S8	PIV3	PIV3	PIV3	-	PIV3
S9	PIV3	PIV3	PIV3	-	PIV3
S10	PIV4	PIV4	None	PIV4	PIV4
S11	PIV4	PIV4	PIV4	-	PIV4
S12	BOCA	None	None	-	None
S13	OC43	HKU1	HKU1	OC43	None
S14	OC43	OC43, RV/EV	OC43	-	OC43
S15	RSVA	RSV	RSV	-	RSV
S16	RSVA	RSV	RSV	-	RSV
S17	NL63	NL63	None	NL63	NL63
S18	229E, HRV	229E, RV/EV	RV/EV	229E, HRV	229E, RV/EV
S19	229E, RSVB	229E, RV/EV, RSV	229E, RSV	-	229E, RSV
S20	NL63	NL63	NL63	-	NL63
S21	MPV	MPV	MPV	-	MPV
S22	BOCA	None	None	-	None
S23	HRV, HEV, PIV1, RSVA, BOCA	RV/EV, PIV1, RSV	RV/EV, PIV1, BOCA	HRV, HEV, PIV1, RSVA	RV/EV, PIV1, RSV
S24	ADV, HRV, PIV2, PIV3, PIV4, BOCA	ADV, MPV, RV/EV, PIV2, PIV3, PIV4	ADV, RV/EV, PIV3	ADV, HRV, HEV, PIV2, PIV3, PIV4	ADV, RV/EV, PIV2, PIV3, PIV4
S25	BOCA	PIV2	PIV2, BOCA	-	PIV2
S26	ADV, BOCA	ADV, HKU1	ADV	-	ADV
S27	BOCA	None	None	-	None
S28	FLUA	FLUA/H1-2009	FLUA/H1-2009	-	FLUA
S29	FLUA	FLUA/H3	FLUA/H3	-	FLUA
S30	ADV	ADV	ADV	-	ADV
S31	FLUA	FLUA/H1-2009	FLUA/H1-2009	-	FLUA
S32	MPV	MPV	MPV	-	MPV
S33	MPV	ADV, MPV	ADV, MPV	-	ADV, MPV
S34	PIV3	PIV3	PIV3	-	PIV3
S35	FLUA	FLUA/H3	FLUA/H3, PIV1	-	FLUA
S36	NL63	NL63	NL63†	-	NL63
S37	PIV4	None	PIV4	PIV4	PIV4
S38	OC43	OC43	None	OC43	OC43
S39	OC43	OC43	None	OC43	OC43
S40	PIV2	PIV2	PIV2	-	PIV2
S41	ADV, OC43, HEV, RSVB	ADV, OC43, RV/EV, RSV	ADV, OC43, RV/EV, RSV	-	ADV, OC43, RV/EV, RSV
S42	HEV, FLUA, PIV4	FLUA/H3, PIV4	FLUA/H3	FLUA, PIV4	FLUA, PIV4
S43	PIV3	RV/EV, PIV3	PIV3	-	PIV3
S44	OC43, MPV	OC43, MPV, RV/EV	OC43, MPV	-	OC43, MPV
S45	RSVB	RSV	RSV	-	RSV
S46	MPV	MPV	MPV	-	MPV
S47	NL63	NL63	RSV	NL63	NL63
S48	ADV	ADV	None	ADV	ADV

229E, coronavirus 229E; ADV, adenovirus; ARV, Seegene Anyplex™ II RV16; BOCA, bocavirus; FARP, BioFire^®^ FilmArray^®^ Respiratory 2.1 plus Panel; FLUA, influenza A; FLUB, influenza B; HEV, human enterovirus; HKU1, coronavirus HKU1; HRV, human rhinovirus; MPV, metapneumovirus; NL63, coronavirus NL63; OC43, coronavirus OC43; PIV1, parainfluenza virus 1; PIV2, parainfluenza virus 2; PIV3, parainfluenza virus 3; PIV4, parainfluenza virus 4; QRP, Qiagen^®^ QIAstat-Dx^®^ Respiratory SARS-CoV-2 Panel; RSV, respiratory syncytial virus; RSVA, respiratory syncytial virus A; RSVB, respiratory syncytial virus B; RV/EV, rhinovirus/enterovirus.

† , QRP repeated due to invalid run (cartridge failure).

**TABLE 2:** Performance of the Seegene Anyplex™ II RV16, BioFire^®^ FilmArray^®^ Respiratory 2.1 *plus* Panel, and Qiagen^®^ QIAstat-Dx^®^ Respiratory SARS-CoV-2 Panel tested on 48 respiratory samples collected in South Africa during 2021 and 2022.

Molecular Assay	True positives (*n*)	True negatives (*n*)	False positives (*n*)	False negatives (*n*)	Overall sensitivity %	Overall specificity %
ARV (*N* = 720)	57	660	1	2	96.6	99.8
FARP (*N* = 768)	56	704	7	1	98.2	99.0
QRP (*N* = 768)	46	709	2	11	80.7	99.7

ARV, Seegene Anyplex™ II RV16; FARP, BioFire^®^ FilmArray^®^ Respiratory 2.1 plus Panel; QRP, Qiagen^®^ QIAstat-Dx^®^ Respiratory SARS-CoV-2 Panel.

Results of 27 specimens (56.3%) were in consensus across all three assays, while the remaining specimens produced discordant results that amounted to 14 FN and 10 FP results. The QRP contributed 11 FN results, of which five involved the coronaviruses and four the parainfluenza viruses. Target-specific sensitivities for coronaviruses 229E, OC43 and NL63 were equal to or less than 60.0%. Five of the seven FP results on the FARP were due to the rhinovirus/enterovirus target, which translated to a target specificity of 88.4%. However, a McNemar’s chi-squared test showed no statistically significant impact on FARP performance (*p* = 0.07).

The sensitivity and specificity for both influenza A and influenza B viruses were 100.0%. All seven specimens with a composite reference for influenza A were subtyped by the FARP and QRP, apart from one where the FARP subtyped it as H1-2009, but the QRP failed to subtype the detected influenza A target.

## Discussion

This study evinces reliable accuracy of the ARV and FARP when compared to the CRS, but calls attention to the sensitivity of the QRP. It adds to the body of performance characteristics described for respiratory molecular panels and is, to the authors’ knowledge, the first comparison of the latest versions of these assays in Africa. The specimens used for this study were selected to include a diverse range of comparable targets to best assess the performance of the platforms. The overall accuracy of the ARV and FARP were found to be comparable, but the QRP demonstrated lower sensitivity.

The high overall sensitivity (98.2%) achieved by the FARP contrasts with the overall sensitivity of 84.5% with other platforms that was found in a comparative study in 2012 from North Carolina in the United States of America, which also demonstrated significantly lower sensitivity (57.1%) for the detection of adenovirus.^4^ Our study revealed a 100.0% sensitivity for adenovirus, whereas both the ARV and QRP showed sensitivities of 83.3% for this target. It was suggested that certain adenovirus serotypes were missed by the previous iterations of the FARP, but since the implementation of version 1.7 (current version is 2.1 *plus*), retrospective and prospective studies have shown improved adenovirus sensitivity as demonstrated in 2013 at a paediatric department in Texas, United States of America.^25^ Five FP results were identified for the FARP, which involved the detection of the rhinovirus/enterovirus target. This generated the lowest target-specific specificity (88.4%) for any platform, and even though this was not statistically significant in comparison to the CRS (*p* = 0.07), the possible impact of the small study sample should be highlighted. The lack of statistical difference between the FARP and CRS specificities seems substantiated as no literature was found in support of high FP rates for this target.

The ARV achieved the best overall specificity compared to the other two platforms, while its overall sensitivity was also acceptable as per South African National Accreditation System criteria. A previous study from South Korea recommended in 2013 that the sensitivity for the detection of human rhinovirus (88.8%) required improvement.^26^ However, this was not found in our study, since the ARV sensitivity (and specificity) for this target was 100.0%.

A peculiar finding in one specimen was the detection of coronavirus OC43 by the ARV, but coronavirus HKU1 by both the FARP and QRP. According to the composite reference methodology, coronavirus HKU1 would have been the standard reference. However, this target was excluded from analysis as the ARV assay does not detect HKU1. This apparent misidentification was noted by another comparative study from 2018 that was conducted in a general hospital of Singapore, but not described in further detail.^5^ The study further commented that distinguishing coronavirus subtypes may be clinically irrelevant as they were historically accepted to cause mild disease and were not monitored for circulation in the population.^5^ However, this perception has been challenged in the last two decades by the identification of risk groups for severe disease^27^ and, more recently, refuted by the spectrum of pathology caused by SARS-CoV-2 during the coronavirus disease 2019 pandemic.^28^

Although nucleic acid integrity was a concern due to the frozen storage of specimens after routine ARV runs, only two from a total of 58 positive targets (3.4%) failed detection in the repeat runs, and thus seemed to have degraded. It was noted though that of the seven bocavirus targets that were detected by the ARV, only two were detected by the QRP. These specimens were not all retested by the ARV. It remains unclear whether the QRP has a lower sensitivity for bocavirus, or whether failed detection was due to nucleic acid degradation.

Contrary to previous evaluation studies of the QRP in Germany (2020) and France (2021), its overall sensitivity in this study (80.7%) did not meet South African National Accreditation System acceptance criteria.^6,28^ This was particularly evident among the coronavirus, and to a lesser extent, the parainfluenza virus targets. Although one would be inclined to label these targets as problematic based on this study, the few numbers of specimens analysed during this study remains a point to scrutinise. Whether these pathogens contribute to severe illness, as mentioned previously, is another consideration. Notably, the ARV and FARP yielded no FN results for the coronavirus targets. The other QRP FN results were for adenovirus and respiratory syncytial virus.

The two FP results of the QRP included parainfluenza virus 1 and respiratory syncytial virus. It was noted that the cycle thresholds values were 33.0 for parainfluenza virus 1 and 31.4 for respiratory syncytial virus. These are higher than the median cycle thresholds value of the true positive results (25.7). This implies that the ARV and FARP may have missed these targets and that more extensive discrepancy testing could have been of benefit.

As the purpose of these assays is detecting pathogens, the major concern of the QRP sensitivity noted in this study cannot be disregarded. But it would be remiss not to recommend that validations of such assays with an appropriate cohort number should be conducted in each laboratory to ensure accurate results.

### Limitations

The sample size of this study was limited by the number of kits sponsored by the manufacturers due to supply challenges during the coronavirus disease 2019 pandemic. Processing of both routine and thawed specimens occurred which might confound comparison of targets, especially where nucleic acid degradation could have transpired. Even though care was taken to account for specimen integrity, it was not within the scope of this study to resolve discrepancies definitively. Additionally, as the specimens constituted a selected population (and not a sample of a particular population), the sensitivities and specificities were precise and therefore confidence intervals were not applicable. Important targets that were not assessed include SARS-CoV-2 and the bacterial pathogens – exclusion was due to the routine assay (ARV) not being able to detect these targets, and making use of single plex assays to detect them was beyond the scope of this research. As most pathogens were studied in small numbers, extrapolation of the performances of the assays should not be strictly applied.

### Conclusion

As multiplex molecular platforms are gaining popularity within clinical diagnostics, rigorous verification of their performance should be underscored. This study demonstrated comparable sensitivity and specificity of the ARV and FARP using a CRS on overlapping targets of stored respiratory specimens. Although the QRP produced comparable specificity, its sensitivity was inferior. A more extensive prospective study is required to assess additional targets over a larger sample size.
